# Facile tuning of the mechanical properties of a biocompatible soft material

**DOI:** 10.1038/s41598-019-43579-8

**Published:** 2019-05-09

**Authors:** Daniele Vigolo, Shivaprakash N. Ramakrishna, Andrew J. deMello

**Affiliations:** 1 Institute for Chemical and Bioengineering, Department of Chemistry and Applied Biosciences, ETH Zurich, Vladimir Prelog Weg 1, Zürich, 8093 Switzerland; 2 Laboratory for Surface Science and Technology, Department of Materials, ETH Zürich, Vladimir-Prelog-Weg 5, CH-8093 Zürich, Switzerland; 3 Present Address: School of Chemical Engineering, University of Birmingham, Edgbaston, Birmingham B15 2TT UK

**Keywords:** Biomaterials, Gels and hydrogels, Biomedical engineering

## Abstract

Herein, we introduce a method to locally modify the mechanical properties of a soft, biocompatible material through the exploitation of the effects induced by the presence of a local temperature gradient. In our hypotheses, this induces a concentration gradient in an aqueous sodium alginate solution containing calcium carbonate particles confined within a microfluidic channel. The concentration gradient is then fixed by forming a stable calcium alginate hydrogel. The process responsible for the hydrogel formation is initiated by diffusing an acidic oil solution through a permeable membrane in a 2-layer microfluidic device, thus reducing the pH and freeing calcium ions. We characterize the gradient of mechanical properties using atomic force microscopy nanoindentation measurements for a variety of material compositions and thermal conditions. Significantly, our novel approach enables the creation of steep gradients in mechanical properties (typically between 10–100 kPa/mm) on small scales, which will be of significant use in a range of tissue engineering and cell mechanosensing studies.

## Introduction

Gradients of mechanical properties are found in innumerable natural systems and phenomena and at all length scales^1–3^. The behaviour of cells cultivated on substrates of different stiffness have been widely studied in recent years, unveiling how migration^4–6^, proliferation^6,7^, adhesion^5,8^ and also differentiation^7,9,10^ are dependent on the characteristics of the material upon which the cells proliferate. To date most of these studies have considered materials of defined but uniform rigidity, with few assessing biocompatible materials possessing a rigidity gradient^11–13^. This is relevant, for example, for the osteotendinous insertion, the tissue formed by differentiations of Mesenchymal Stem Cells (MSCs) that connects the soft tendinous and the rigid osseous tissue. This is characterised by a gradient of stiffness responsible for the differentiation of the stem cells^14^. In this respect, control over the Young’s modulus of such materials is extremely limited. Herein, we show for the first time the creation, tuning and control of mechanical property in biocompatible materials by inducing a concentration gradient through the application of thermophoretic forces. Significantly, the ability to impose and control temperature gradients across a microfluidic channel allows facile manipulation of elasticity gradients in the final material.

Two major challenges faced when generating rigidity gradients relate to the availability of polymers that permit the formation of the desired gradient in mechanical property and the accessibility of formation methodologies. Unsurprisingly, both issues are closely related, with biocompatibility requirements further complicating the undertaking.

Traditionally, the rigidity of a gel can be tuned by controlling the degree of crosslinking or the initial monomer concentration. For example, the use of an optical mask containing a spatial gradient in transparency can be used to control light intensity during the photo-polymerization process^12^. Alternatively, a microfluidic gradient generator^15^ and a photo-polymerization step can be used to “fix” a desired concentration gradient within a material. Nevertheless, the main limitations of such techniques relate to the choice of the substrate material, which is restricted to UV curable polymers, and reduced control over the magnitude of the rigidity gradient.

To address the aforementioned limitations, we herein exploit the possibility to accurately impose and control a temperature gradient within a microfluidic device to create a novel class of biocompatible material presenting a rigidity gradient, based on crosslinked calcium alginate constrained within microfluidic geometries. In the presence of a temperature gradient, the solutes dispersed in solution are affected by thermophoresis which induces the formation of a concentration gradient. Here we suggest a mechanism based on the combined effect of the thermophoretic drift of each component dispersed in the aqueous solution of sodium alginate to explain the obtained results. Significantly, since thermophoresis can be applied to any kind of solute dispersed in solution, the basic method can be easily extended to any polymer or hydrogel undergoing a polymerization or sol-gel process. The main advantage of our proposed approach is the fact that we can effectively decouple the control over the local stiffness of the material from the polymerisation process. By exploiting thermophoresis, we can induce a gradient of mechanical properties that is independent from the choice of the material. For example, the concentration gradient can be gently tuned by thermophoresis in a delicate biocompatible hydrogel using only minute temperature differences that do not alter the overall properties of the material and then, independently, the material can be crosslinked.

Thermophoresis is a physical phenomenon discovered over one century ago^16,17^. Briefly, in the presence of a temperature gradient, a solute dispersed in solution will migrate along that temperature gradient and accumulate either on the ‘cold’ or the ‘hot’ side depending on the specific solute-solvent interactions^18^, average temperature^19^, solute size^20,21^ or type of dispersed electrolyte^22^. At steady-state, the mass flux, *J*_*m*_, is zero and the concentration gradient is counterbalanced by the temperature gradient (Eq. 1), where the gradient is assumed to be along the z-axis, i.e.

1 $$\begin{array}{ccc}{J}_{m} & = & \rho [D\frac{dc}{dz}-c(1-c){D}_{T}\frac{dT}{dz}]\\ {J}_{m} & = & 0\to \frac{dc}{dz}=-\,c(1-c){S}_{T}\frac{dT}{dz}\end{array}$$

here, *ρ* is the density of the solute, *c* is the concentration, *D* is the diffusion coefficient, *D*_*T*_ is the thermophoretic mobility and *S*_*T*_ the so called Soret coefficient, equal to *D*_*T*_/*D*. In simple terms, the Soret coefficient is positive when solute accumulates on the low temperature side and negative when accumulation occurs on the high-temperature side.

The concentration gradient induced by thermophoresis is proportional to the temperature gradient and, as a consequence, at small scales typical of microfluidic devices, only a very limited temperature difference is required over the biocompatible material to generate the required mechanical properties gradient, and no other material property is influenced by this.

## Results

Imposition of a temperature gradient allows the creation and maintenance of a concentration gradient in a solution of monomers, such as the chains of sodium alginate used in the present study. Controlled diffusion of di- or tri-valent ions (e.g. Ca^2+^, Fe^3+^ and Al^3+^)^23^ can then be used to “fix” the final configuration. Significantly, temperature gradients can be easily generated and controlled within microfluidic environments (for example by embedding Joule heaters^24–26^), with the manipulation of complex fluids in such geometries leading to precisely controlled substrate properties.

### Microfluidic fabrication of calcium alginate substrate

**Figure 1:**
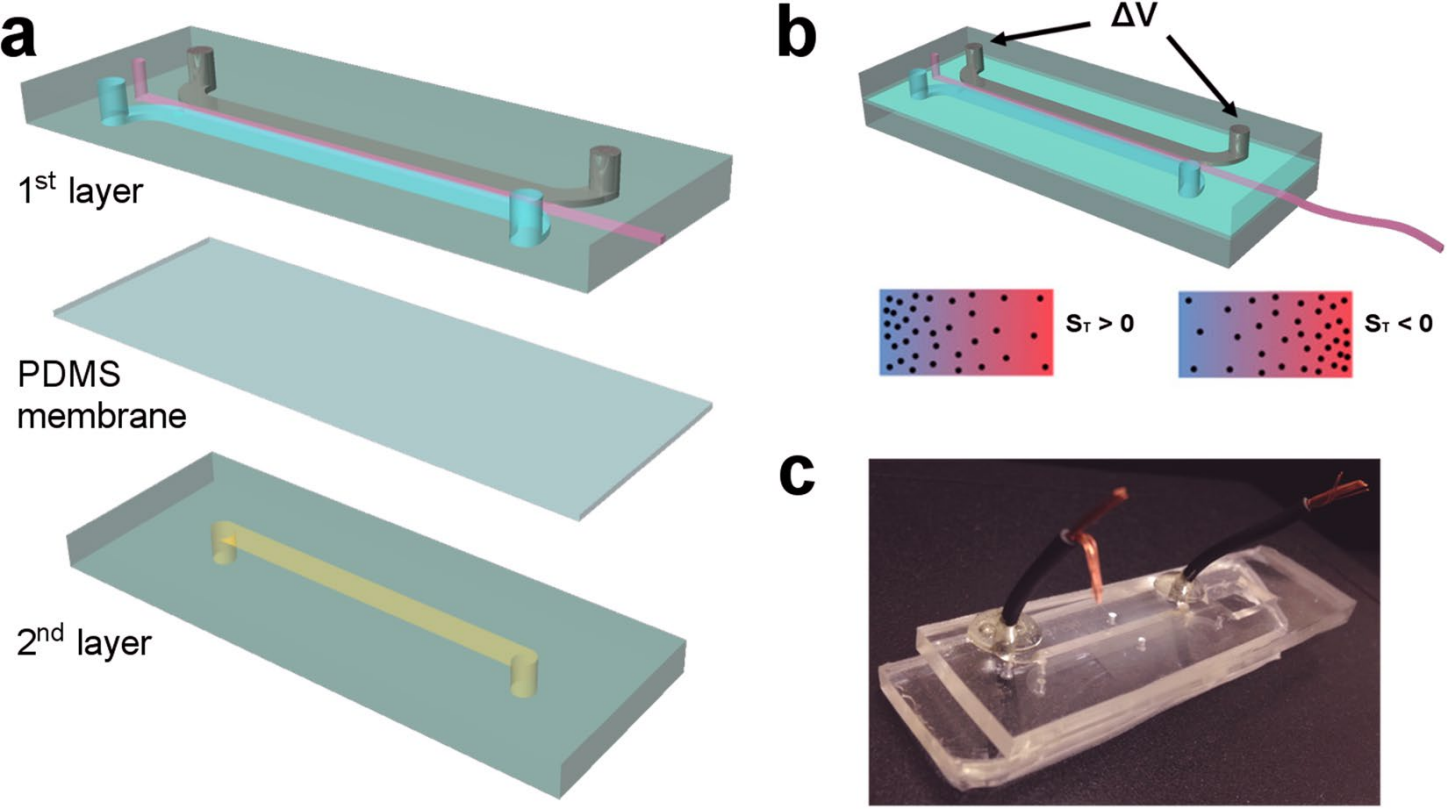
Device schematic. (**a**) Schematic of the two-layer device. (**b**) The working principle behind thermophoresis: a temperature gradient is imposed transversally in the channel, with sodium alginate molecules migrating along the temperature gradient and generating a concentration gradient responsible, which in turn creates a gradient in mechanical properties of the final biocompatible material. (**c**) Photograph of the device used in the current experiments.

As previously noted, the concentration gradient material was formed in a two-layer PDMS (polydimethylsiloxane) microfluidic device integrating a thin PDMS membrane. In the first layer a solution of sodium alginate, a dispersion of 80 nm diameter calcium carbonate nanoparticles and 200 nm diameter polystyrene beads (used to visualize the hydrogel) were delivered manually using a syringe connected via a 0.020″ × 0.060″ OD Tygon microbore tubing (Cole-Parmer Instrument Co. Ltd., UK) into a 600 μm wide and 70 μm (or 140 μm) deep microchannel. Once the flow inside the microchannel was completely stopped, a transverse temperature gradient was then imposed by exploiting the presence of two larger parallel channels (2 mm wide) that flank the main microchannel. One of these larger channels was filled with a low melting point alloy (LMPA) heated above its melting temperature (to allow entry) and then cooled to ambient to realize a conductive path that acts as a Joule heater. In the other flanking channel, cold water was motivated at a volumetric flow rate in excess of 300 μL/min to ensure efficient removal of heat from the sample. On the second layer a 2 mm wide and 140 μm thick microchannel was aligned with the primary microchannel in the first layer (Fig. 1) and filled with an acidic oil solution (acetic acid 5% v/v in soybean oil). Diffusion through the membrane, provides an efficient route to lowering the pH of the sample and dissolving the calcium carbonate nanoparticles that free Ca^2+^ ions and ultimately prompt the formation of the calcium alginate hydrogel. Details regarding the fabrication of the device and all sample preparation methods are provided in the experimental section.

To assess our ability to control the formed concentration gradient, we performed a series of experiments at different average temperatures, whilst maintaining a transversal temperature gradient of between 0 and 8 °C/mm across the microchannel containing the sodium alginate solution for between 20 and 60 minutes. Gelation, induced by the acidic oil solution, took place within 2 to 5 minutes, with the calcium alginate hydrogel being easily extruded through the manual application of pressure by means of a syringe containing water connected to the inlet of the main microchannel. At the outlet, a small chamber filled with water was used to receive and collect the extruded material. Water is ideal in this respect, since it prevents the hydrogel dehydration and assists hydrogel floatation. The sample is then collected together with water and stored at 4 °C prior to AFM nano-indentation experiments.

We hypothesise that sodium alginate migrates along the temperature gradient with a characteristic time that is dependent on its mass diffusion coefficient, *D*, being the latter proportional to its characteristic size via the Stokes-Einstein equation,

$$D=\frac{kT}{6\pi \eta R},$$

where *k* is the Boltzmann constant, *T* the temperature, *η* the viscosity and *R* the hydrodynamic radius. Due to the difficulty in optically visualizing sodium alginate molecules, we assumed that they were affected by thermophoresis in the range of temperature we explored. Their migration dynamics in the presence of a temperature gradient was estimated through the thermophoretic behaviour of fluorescently labelled polystyrene particles (having an average diameter of 200 nm) dispersed in solution. Being alginate molecules much smaller than 200 nm (we estimated the alginate molecule size used in our experiments to be between 5 and 7 nm – see ESI for more details), they will migrate much faster than the polystyrene particles. Based on this analysis, the average time required for the polystyrene particles to start to drift appreciably within the temperature gradient was 45 minutes (Fig. S1). By ensuring that all measurements were performed before this characteristic time, the formation of a polystyrene particle concentration gradient was avoided and the gradient of mechanical properties induced by the calcium alginate concentration gradient could be evaluated in an unambiguous manner. It is worth noticing that besides sodium alginate, also CaCO_3_ particles will be affected by thermophoresis. The characteristic diffusion time is proportional to the particle size and so, the minimum time for the calcium carbonate particle to migrate can be estimated as the calculated minimum time required for the polystyrene particles to migrate times R_CaCO3_/R_PS_, where R_CaCO3_ is the radius of the calcium carbonate particles (40 nm) and R_PS_, is the radius of the polystyrene particles (100 nm). As a result, for experiments shorter than 18 minutes only sodium alginate molecules are responsible for the concentration gradient and so for the stiffness gradient, for longer experiments a weak concentration gradient of calcium carbonate nanoparticles will also affect the final mechanical properties gradient^27^. Finally, once the calcium carbonate nanoparticles are dissolved, also the Ca^2+^ ions will be affected by the temperature gradient and will eventually create a concentration gradient of calcium ions that could also affect the overall stiffness gradient. The concentration gradient of calcium ions will be much weaker than the concentration gradient of alginate molecules as calcium ions have only 2 to 5 minutes to migrate while the viscosity is increasing due to the ongoing crosslinking process, and they present a much smaller *S*_*T*_^28^. Nonetheless, due to the possibility of an “ion cage effect” induced by the sodium alginate molecules, we cannot exclude a more complex local distribution of calcium ions.

Although currently we cannot quantify experimentally the relative concentration gradients of each component, our results demonstrated that the stiffness gradient is solely dependent on (and proportional to) the magnitude of the temperature gradient imposed and the average temperature (see Fig. 3b where we also showed that an identical material generated at uniform temperature does not present a stiffness gradient).

**Figure 2:**
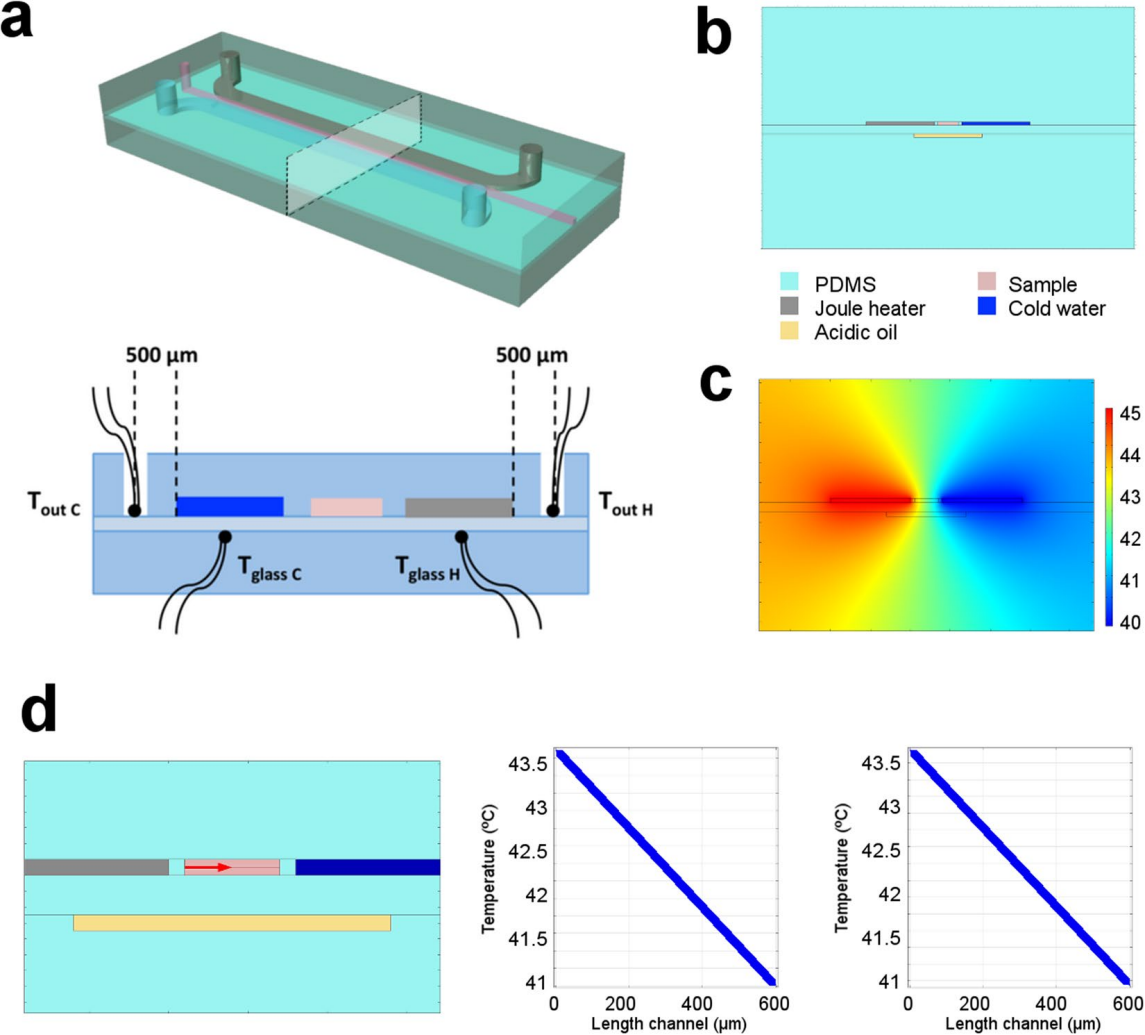
On-chip temperature evaluation. (**a**) Cross section considered for temperature measurements and location of the relative positions of the thermocouples used to calibrate the temperature in real time. (**b**) Geometry used for the 2D numerical simulations. (**c**) Distribution of temperature at steady state, as obtained for the case *T*_*hot*_ = 45 °C and *T*_*cold*_ = 40 °C. (**d**) Temperature gradients across the channel length. In the schematic, the red horizontal arrow indicates the direction along which the temperature gradient is evaluated. The two graphs report the gradient of temperature inside the channel in the device with the PDMS membrane (left) and in the dummy device (right). In both cases the gradient is linear, with difference between the two situations being negligible.

To characterize differing thermal conditions and relative concentration gradients, temperature must be monitored in a robust and precise manner. Since measurement of the temperature gradient of an actual sample is arduous, we adopted an indirect method, where the temperature measured beside the heater and cooler was used to estimate the actual temperature of the sample using calibration measurements obtained from a dummy device, in which the PDMS membrane is substituted by a glass coverslip to improve thermal conductivity (Fig. 2). Additionally, we performed 2D numerical simulations (COMSOL Multiphysics 4.4a, Massachusetts, USA) to evaluate the cross-sectional distribution of temperature in the middle plane of the device (Fig. 2).

### Assessment of local mechanical properties by atomic force microscopy

**Figure 3:**
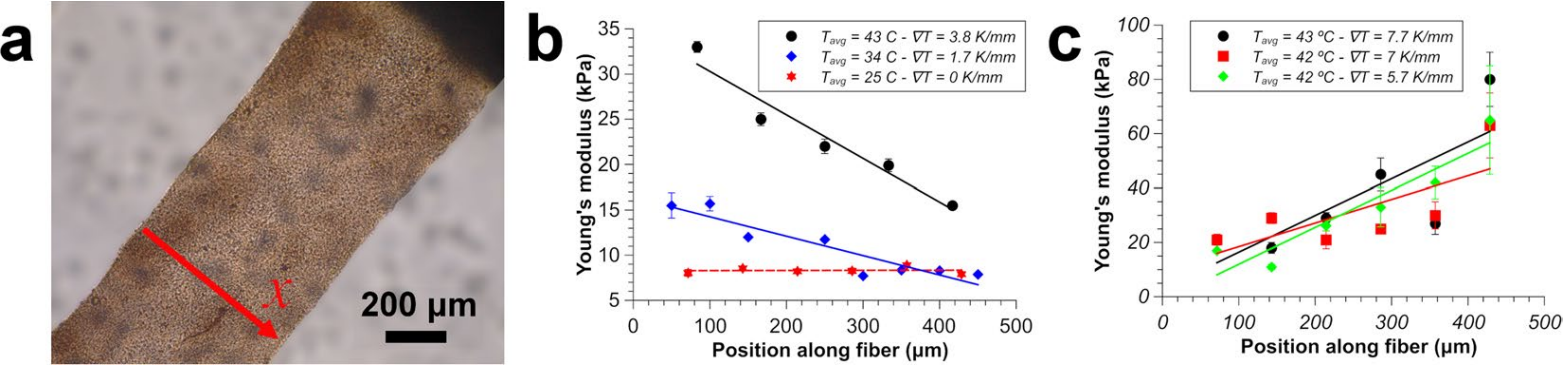
Characterization of the mechanical properties of the concentration gradient biocompatible material. (**a**) An optical image of the material under test. The red arrow indicates the direction of measurement. (**b**) Variation of the Young’s modulus as a function of position along the fibre for a 1% w/v sodium alginate sample. (**c**) Variation of the Young’s modulus as a function of position along the fibre for three samples prepared using an initial concentration of 0.6% of sodium alginate. For similar average temperatures, and a temperature gradient of more than 5 °C/mm, the gradient in mechanical properties is broadly similar. Solid lines indicate linear fits to the experimental data. Error bars represent standard errors.

After sample collection, the mechanical properties (in particular the Young’s modulus) were measured using AFM-based nanoindentation to assay the elasticity of the biocompatible material in the transverse direction immersed in water (Fig. 3). A silicon (100) wafer (Si-Mat Silicon Wafers, Germany) was cut into 2 × 1 cm pieces and cleaned by 15 minutes of sonication in ethanol followed by 30 minutes of UV ozone treatment (UV Ozone Cleaner – ProCleaner, BioForce Nanosciences, USA). The negatively charged silicon wafers were then immersed in a solution of 1 mg/ml of poly(ethyleneimine), which renders the surface positively charged. The prepared calcium alginate concentration gradient material contains a residual negative charge that ensures good adhesion to the silicon substrate during such indentation measurements.

Assuming that the local concentration of sodium alginate molecules was the main contribution to the stiffness gradient, we evaluated the sign of *S*_*T*_, and thus the region in which sodium alginate molecules accumulate. Calcium alginate fibres were carefully extruded and attached directly on the silicon substrate in a manner that preserves their orientation. This allows establishment of the direction of the thermophoretic drift, based on the hypothesis that the “stiffer” side of the material corresponds to the region where sodium alginate molecules accumulate. These experiments indicated that, within the temperature range explored and in the presence of calcium ions, sodium alginate always accumulates on the “colder” side, meaning that the associated Soret coefficient is positive.

Indentation measurements were performed using a silica colloidal probe, with the sample completely submerged in deionized water to avoid dehydration of the hydrogel during measurement. The use of a colloidal probe provided a well-defined geometry and reduced contact pressures on indentation. Figure 3 illustrates how the temperature gradient induces an appreciable elasticity gradient that can be controlled by tuning not only the magnitude of the temperature gradient but also the average temperature. It should also be noted that since PDMS does not prevent water evaporation, the sodium alginate sample tends to dehydrate slightly during the experiments, especially at higher temperatures, resulting in an increase of the average alginate concentration. This is likely to be the reason why, despite the fact that samples were prepared simultaneously for all three experimental conditions, the average elasticity is higher for the sample prepared at an elevated temperature.

## Discussion

We have demonstrated for the first time a direct method for modifying the mechanical properties of a soft biocompatible material by leveraging a temperature gradient imposed across a microfluidic channel. The technique relies on the establishment of thermophoretic forces, and thus defines a generic method applicable to a wide range of related materials. Specifically, we were successful in inducing a 6-fold variation in Young’s modulus across a micron-scale substrate by establishing a temperature variation of less than 5 °C across the microchannel width. Significantly, we have also demonstrated precise control of the elasticity gradient by varying both the sample concentration and thermal conditions (such as the temperature gradient magnitude and the average temperature). Finally, extensive AFM nano-indentation measurements were used to validate and characterize our system. We believe that this novel method will significantly impact a range of cell mechanosensing studies, through the expansion of accessible substrate materials and, more importantly, by enabling investigation of cell responses to mechanical property/concentration gradients on the single-cell scale.

## Materials and Methods

### Sample and device fabrication

Alginic acid sodium salt from brown algae, soybean oil, sodium hydroxide and acetic acid were obtained from Sigma Aldrich, Switzerland, and used as received. Fluorescent polystyrene particles (Molecular Probes FluoSpheres, with an average diameter of 200 nm) were purchased from Thermo Fisher Scientific, USA. Calcium carbonate nanoparticles (with an average diameter of 80 nm) were purchased from MKnano, Canada. Low melting point alloy (MCP 96/Metspec 203 Alloy or Rose’s “A” alloy, 5N Plus, Germany), comprising Bi 54%, Pb 28%, Sn 18% was obtained as a gift from 5N Plus, Germany. Two sodium alginate aqueous solution concentrations (0.6 and 1% w/v) were used in the current experiments. Their pHs were varied between 8 and 8.2, through addition of NaOH. An alkaline solution was used to ensure that dispersed calcium carbonate particles did not dissolve during the course of an experiment. Concentrations of polystyrene and calcium carbonate nanoparticles were kept constant for all the samples prepared at 0.2% w/v and 22 mM respectively. The acidic oil solution was prepared by adding 5% v/v of acetic acid to soybean oil.

Top and bottom PDMS (Sylgard™ 184 Silicone Elastomer Kit, Dow Corning, UK) layers were prepared using standard soft-lithographic techniques. Briefly, a silicon wafer mold was prepared by exposing a 70 μm or 140 μm thick spin coated layer of SU-8 2075 photoresist (MicroChem, Westborough, USA) resin to UV light through a high precision photomask (Micro Lithography Services Ltd, UK). After development, the negative mold was used to cast PDMS layers by pouring a mixture of PDMS and its curing agent in a 10:1 ratio over the mold, followed by curing at 70 °C for 2 hours. Subsequently, a spin coater was used to create a 250 μm thick PDMS membrane on a glass slide by spinning at 500 rpm for 30 seconds. After curing the membrane in an oven at 70 °C for 2 hours, the first layer of the final device was attached to the membrane by exposing the assembly to a corona discharge (BD-20AC, Electro-Technic Products, USA) and heating it on a hot plate at 100 °C for 20 minutes. The assembly, comprising the first layer and the membrane, was kept on a supporting glass slide and the temperature of the hot plate increased to 120 °C. This allows the low melting point alloy to melt and flow inside one of the large channels. After placing two electrical wires to impose voltage across the Joule heater, the device was left to cool down to ambient. The device was then carefully removed from the supporting glass keeping the membrane intact. Finally, the device was completed by attaching it to the lower PDMS layer by corona treatment, carefully aligning the large channel on the second layer to the main microchannel on the first layer.

### AFM measurements

Indentation measurements were carried out using an atomic force microscope (Asylum AFM, MFP-3D, Oxford Instruments). A silica colloidal sphere (Kromasil, EKA Chemicals AB) with an 8 µm radius was glued to the end of an Au-coated tipless cantilever (Mikromasch) by means of a homebuilt micro-manipulator^29^. The spring constant of the cantilever was calibrated via the thermal-noise method^30^ before attachment of the colloid. Modulus values were estimated from the measured force versus indentation curves using a built-in Hertz fit function within the Asylum Research analysis software suite (version AR12). The indentation depth was limited to less than one third of the sample thickness to avoid any substrate effects in modulus calculations (see ESI for more details). The probe modulus and Poisson ratio were chosen to be 74 GPa and 0.2, respectively. The Poisson ratio for the sample was assumed to be 0.5.

### Temperature evaluation

We initially created a dummy device, where the PDMS membrane was replaced by a thin glass coverslip to improve thermal conductivity. We then placed two small thermocouples (Fine Gauge Exposed Welded Tip Thermocouple - Type K, with 76 μm conductors, Labfacility, UK, purchased at Farnell element14, Switzerland) below each of the two large channels responsible for heating and cooling, and sandwiched these between the glass and the bottom PDMS layer mimicking the real device. We also placed two additional thermocouples 500 μm from the large channels as shown in Fig. 2. Subsequently, we performed a complete set of temperature measurements for different applied voltage and water flow rates. Knowing the thermal properties of each material, the real temperature on the hot and cold side of the microchannel could be estimated by considering the temperature drop across the glass coverslip and across the PDMS layer separating the large channels from the microchannel (details provided in the ESI). Finally, we correlated these temperatures with the values recorded at the two locations 500 μm from the large channels on the outer side. We also performed 2D numerical simulations to confirm that the temperature gradient was not disturbed by the presence of a thin glass slide and was linear (Fig. S2).

### Computational methods

2D numerical simulations were performed using the Heat Transfer Package (for both solids and fluids) in COMSOL Multiphysics 4.4a (Massachusetts, USA). The geometry reflects the one used for the experiments and the presence of a 4 mm thick PDMS layer (modelled as “silicone” in COMSOL) guarantees good insulation of the microchannel from the environment.

## Supplementary information

Electronic Supporting Information

## Data Availability

The datasets generated during and/or analysed during the current study are available from the corresponding author on reasonable request.
