# Supplementary material for: Facile tuning of the mechanical properties of a biocompatible soft material
Source: Sci Rep. 2019 May 9;9:7125. doi: 10.1038/s41598-019-43579-8 (PMC6509115; doi:10.1038/s41598-019-43579-8)
Supplement: Supplementary file 1 — Electronic Supporting Information [file 41598_2019_43579_MOESM1_ESM.pdf]

Facile tuning of the mechanical properties of a biocompatible soft material

By Daniele Vigolo[1,‡,*], Shivaprakash N. Ramakrishna[2], and Andrew J. deMello[1]

[1] Institute for Chemical and Bioengineering, Department of Chemistry and Applied Biosciences, ETH Zurich, Vladimir Prelog Weg 1, Zürich 8093, Switzerland

[2] Laboratory for Surface Science and Technology, Department of Materials, ETH Zürich, Vladimir-Prelog-Weg 5, CH-8093 Zürich, Switzerland

* Corresponding author: d.vigolo@bham.ac.uk ‡ Present address: School of Chemical Engineering, University of Birmingham, Edgbaston, Birmingham, B15 2TT, UK

Electronic Supporting Information – ESI

Evaluation of the concentration gradient of polystyrene nanoparticles

In order to monitor the progress of the thermophoretic drift, we performed experiments evaluating the emission from fluorescently labelled (200 nm) polystyrene particles in the presence of a temperature gradient. Images were analysed according to the method previously described by Vigolo *et al.*,[1] i.e. the average intensity profiles were extracted from each fluorescence image and normalised in order to impose mass conservation. The results are summarized in **Figure S1**, which reports the slope of the linear intensity profile, *dI/dz*, as a function of time. From the plot it is clear that during the first 45 minutes there is no appreciable drift of the polystyrene particles, which confirms that data extracted after a waiting time of between 20 and 45 minutes are solely dependent on the sodium alginate. However, it is observed that for times longer than 45 minutes, migration of polystyrene particles may yield an appreciable change in mechanical properties.

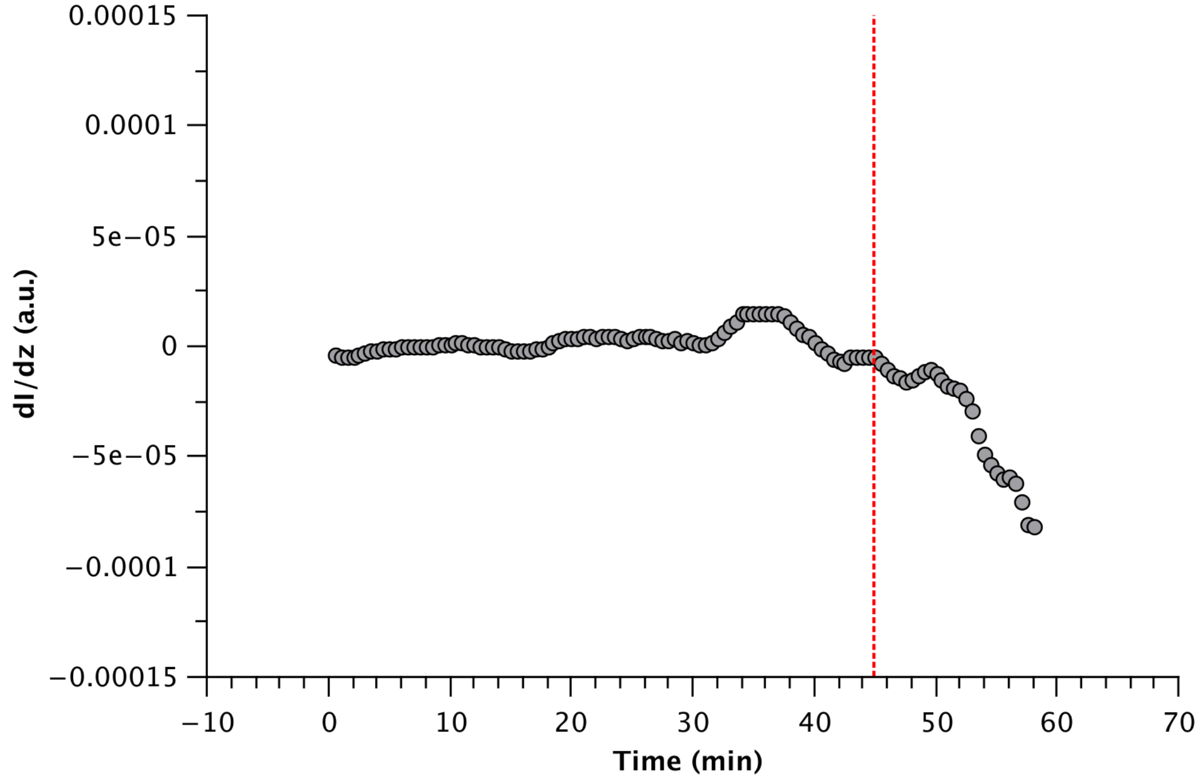

Figure S1. Thermophoresis of polystyrene nanoparticles. The fluorescence intensity, I, of the 200 nm polystyrene particles dispersed in the sodium alginate solution is monitored while a temperature gradient is imposed across the channel. The plot represents the gradient of fluorescent intensity across the channel, dI/dz, versus time. The thermophoretic drift becomes apparent after about 45 minutes. The average temperature was 38.9 ºC and the temperature gradient was 3.3 K mm^{-1}.

Estimation of actual temperature gradient

Temperature is monitored during experiment by two thermocouples placed beside the Joule heater and the cold-water channel (as shown in **Figure 2a**) that record $T_{out\ H}$ and $T_{out\ C}$ respectively. Use of the mock device (described in the main text) allows correlation of these values with the temperatures $T_{glass\ H}$ and $T_{glass\ C}$, measured through a thin glass coverslip directly below the heating and cooling channels. In particular, we experimentally observed that temperatures measured on glass are approximately 1.13 times higher than the temperatures measured beside the bigger channels, since we are always performing experiments at an average temperature that is higher than ambient. This yields the relationship: $T_{glass\ H} = 1.13^{*}\ T_{out\ H}$ and $T_{glass\ C} = 1.13^{*}\ T_{out\ C}$.

Subsequently, we evaluated the actual temperatures of the Joule heater and cold water to assess the influence of the glass coverslip. To do so we performed numerical simulations and evaluated the temperature drop across a 150 μm glass slide below the heater and cooler. The results are presented in **Figure S2** and clearly show that the difference in temperature across the glass is negligible. Accordingly, we consider that the real temperature of the heater and cooler, T_H and T_C respectively, are the same as the temperature measured at the glass side, with $T_{glassH} = T_H$ and $T_{glassC} = T_C$.

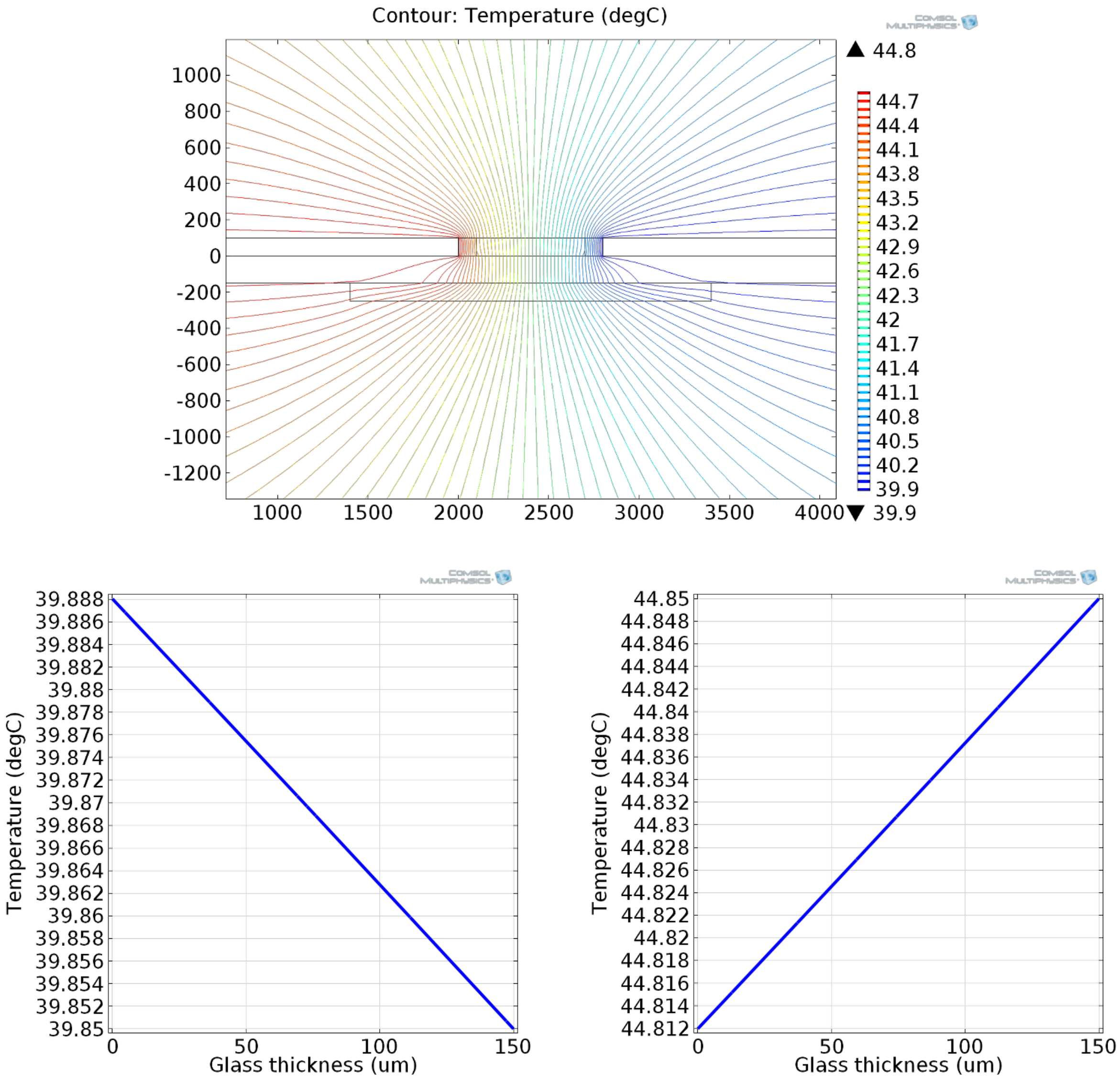

Figure S2. Numerical simulations of the temperature distribution across the glass cover slip in the presence of a temperature gradient. The temperature distribution in the mock device where the PDMS membrane was substituted with a 150 μm thick glass coverslip is shown. The two plots demonstrate the difference in temperature across the thickness of the glass coverslip of respectively the hot and the cold side. Such variation is minimal and can be neglected during the evaluation of temperature.

The temperature across the microchannel can then be estimated through knowledge of the thermal conductivity of all material involved and by applying an electrical analogy model as shown in **Figure S3**.

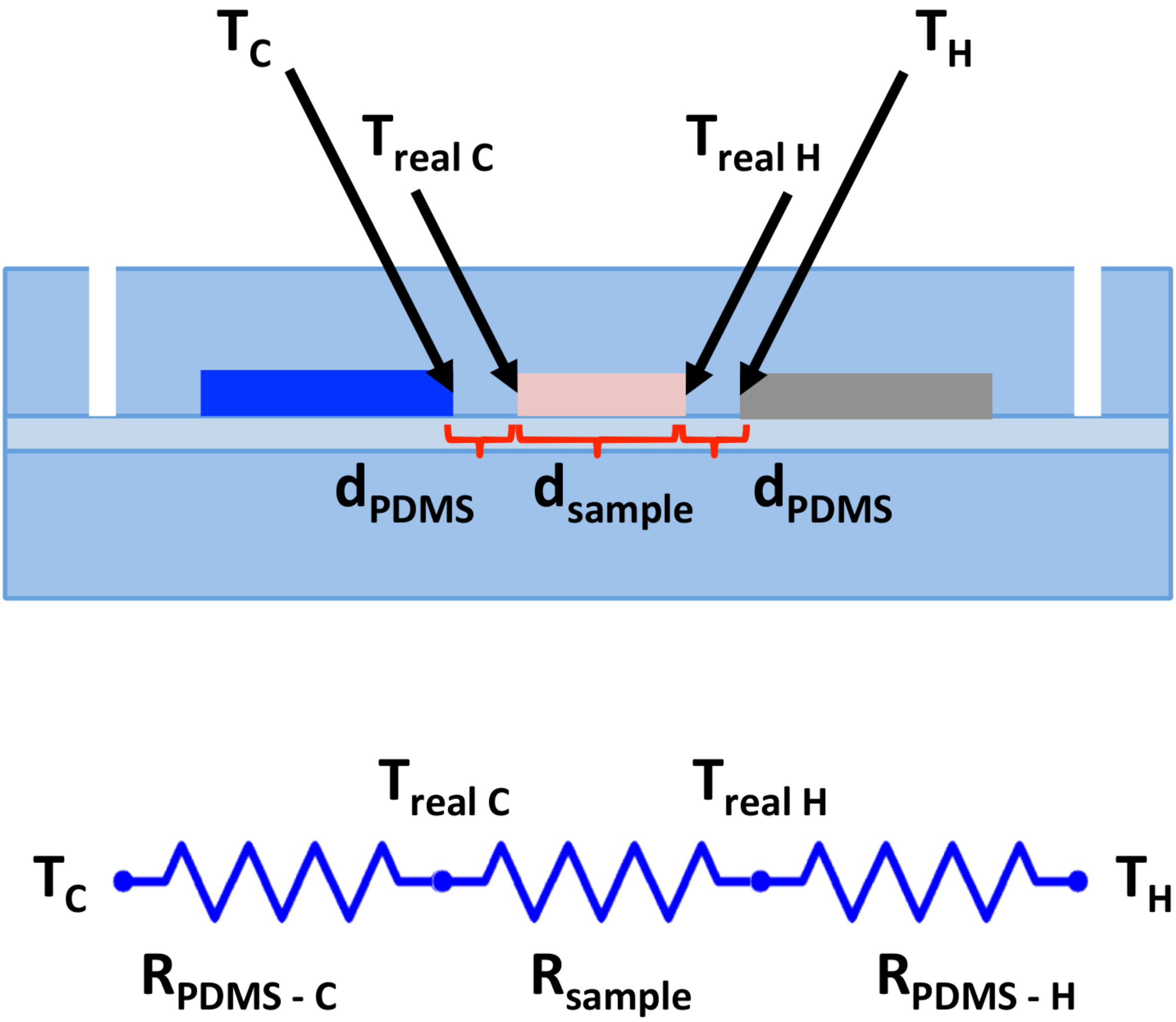

Figure S 3. Schematic of the temperature distribution across the device and its electrical analogy in terms of thermal resistances.

From T_H and T_C, we can estimate the real temperature across the microchannel, ΔT_{sample}, by knowing the thermal conductivities, k, of each material. This relates to the thermal resistivity, R (in the electrical analogy shown in **Figure S3**) as $R = d/k$, where d is the thickness of material. If we define $\Delta T_{tot} = T_H - T_C$, $\Delta T_{PDMS\text{-}C} = T_{real\ C} - T_C$, $\Delta T_{sample} = T_{real\ H} - T_{real\ C}$, $\Delta T_{PDMS\text{-}H} = T_H - T_{real\ H}$, then we can evaluate: $\Delta T_{sample} = \Delta T_{tot} \times R_{sample}/R_{tot}$

Where $R_{sample} = d_{sample}/k_{sample}$, and we consider $k_{sample} = k_{water}$, and $R_{tot} = R_{PDMS\text{-}C} + R_{sample} + R_{PDMS\text{-}H} = 2 \times d_{PDMS}/k_{PDMS} + d_{sample}/k_{water}$.

For all the experiments described we used two different d_{PDMS} in order to obtain different temperature gradient magnitudes.

With $k_{sample} = k_{water} = 0.6$ W m^{-1} K^{-1}, $k_{PDMS} = 0.15$ W m^{-1} K^{-1}, and $d_{sample} = 600$ µm, we obtain for the device with $d_{PDMS} = 100$ µm, $\Delta T_{sample} = \Delta T_{tot} \times 0.43$, and for the device with $d_{PDMS} = 200$ µm, $\Delta T_{sample} = \Delta T_{tot} \times 0.27$. These values were then used to calculate the actual temperature gradient acting transversely on the sodium alginate sample from the measured temperature values detected by the thermocouples.

Estimation of the diffusion coefficient of sodium alginate

The diffusion coefficient of sodium alginate, D, can be estimated knowing its molecular size, the temperature of the sample and its viscosity. Specifically, $D = k_B T/6\pi\eta R$, where k_B is the Boltzmann's constant, η the viscosity and R the effective radius of the sodium alginate molecule. The average molecular size of alginate can be estimated through knowledge of its molecular weight, M, and its density, ρ =1.6 g/cm^3, with its volume, V, being expressed as:

$$V(nm^3) = \frac{\rho(g\,cm^{-3})\times 10^{21}(nm^3 cm^{-3})}{6.023\times 10^{23}(Da\,g^{-1})} \times M(Da) \qquad (2)$$

Accordingly, the equivalent radius, R, is approximately equal to $(3V/4\pi)^{1/3}$. For a single molecule of sodium alginate salt, $C_6H_9O_7Na$ (M = 216.12 Da) we obtain $R = 0.52$ nm. In this context, it is noteworthy to mention that since alginic acid is composed of the β (1→4)-D-mannosyluronic acid (M) and α(1→4)-L-gulosyluronic acid residues (G), and alginate in water is composed of several units of both M and G groups, the final molecular weight could vary between approximately 200 and 600 kDa[2]. Hence, the estimated radius of a chain of alginate in water undergoing thermophoretic drift will vary between 5 and 7 nm.

Another parameter influencing mass diffusion is viscosity. The reported values for the viscosity of a 1% w/w sodium alginate at 25ºC lie between 4 and 12 cps (as stated in the specification sheet of the Sigma-Aldrich sodium alginate used for these experiments). Although the precise determination of the viscosity of sodium alginate is not the purpose of the current study, it is nevertheless worth noting that its exact value is difficult to predict *a priori* as it depends not only on the concentration of sodium alginate, but also on the relative abundances of the *M* and *G* groups, and in general also on the specific batch used to prepare the sample[2].

Evaluation of mechanical properties by AFM

For each sample investigated, Young's moduli were measured at 6 to 8 points along the transverse direction, parallel to the concentration gradient, with each data point representing an average obtained from the evaluation of a matrix of 16 x 2 individual measurements within a 500 x 500nm^2 area.

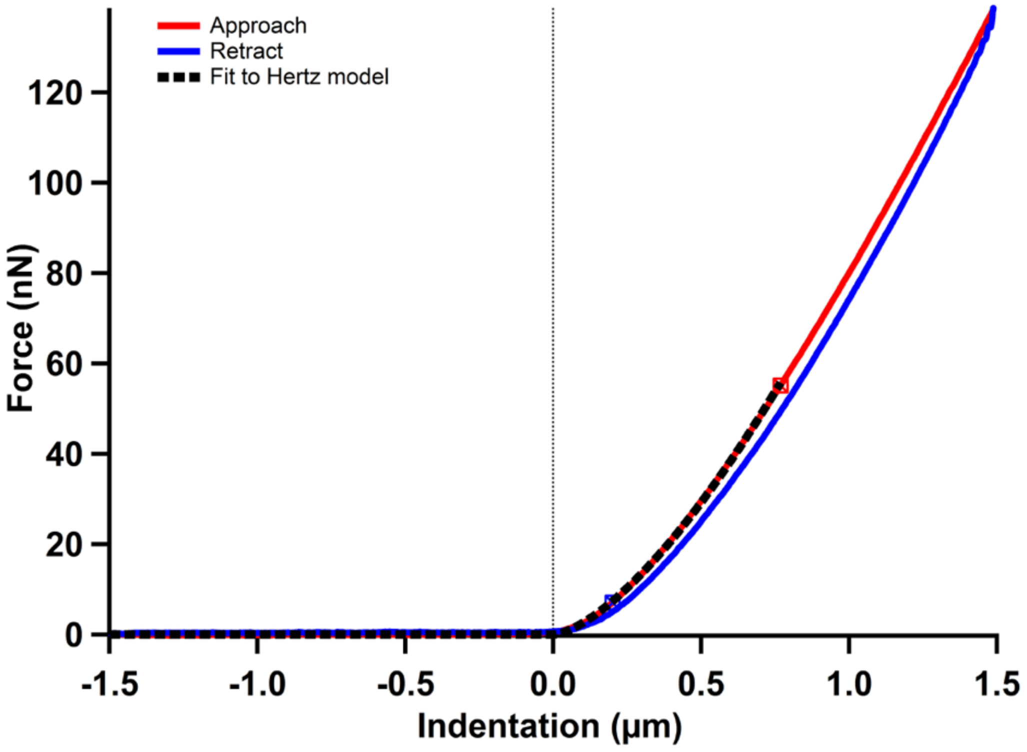

Figure S4. Example of measured force versus indentation curve for a calcium alginate sample prepared with a solution of sodium alginate 1% w/v exposed to a temperature gradient of 3.4 ºC/mm at an average temperature of 43 ºC (the overall elasticity behaviour is shown in Figure 3b of the main text). The red and blue curves represent the approach and retraction curves respectively. The dashed black line is the fit to the Hertz model for the approach curve. The obtained Young's modulus for this particular curve is 16.5 kPa. The spring constant of the cantilever used was 4.57 N/m and the radius of the silica colloidal sphere was 8 µm.

Supplementary References

1. Vigolo, D., Rusconi, R., Stone, H. A. & Piazza, R. Thermophoresis: microfluidics characterization and separation. *Soft Matter* **6,** 3489 (2010).
2. Fu, S. *et al.* Rheological evaluation of inter-grade and inter-batch variability of sodium alginate. *AAPS PharmSciTech* **11,** 1662–74 (2010).
